# Supplementary material for: Enhanced ocean-atmosphere carbon partitioning via the carbonate counter pump during the last deglacial
Source: Nat Commun. 2018 Jun 19;9:2396. doi: 10.1038/s41467-018-04625-7 (PMC6008475; doi:10.1038/s41467-018-04625-7)
Supplement: Supplementary file 1 — Supplementary Information [file 41467_2018_4625_MOESM1_ESM.pdf]

Enhanced ocean-atmosphere carbon partitioning via the carbonate counter pump during the last deglacial

Duchamp-Alphonse* et al.

*corresponding author: stephanie.duchamp@u-psud.fr

Supplementary Information

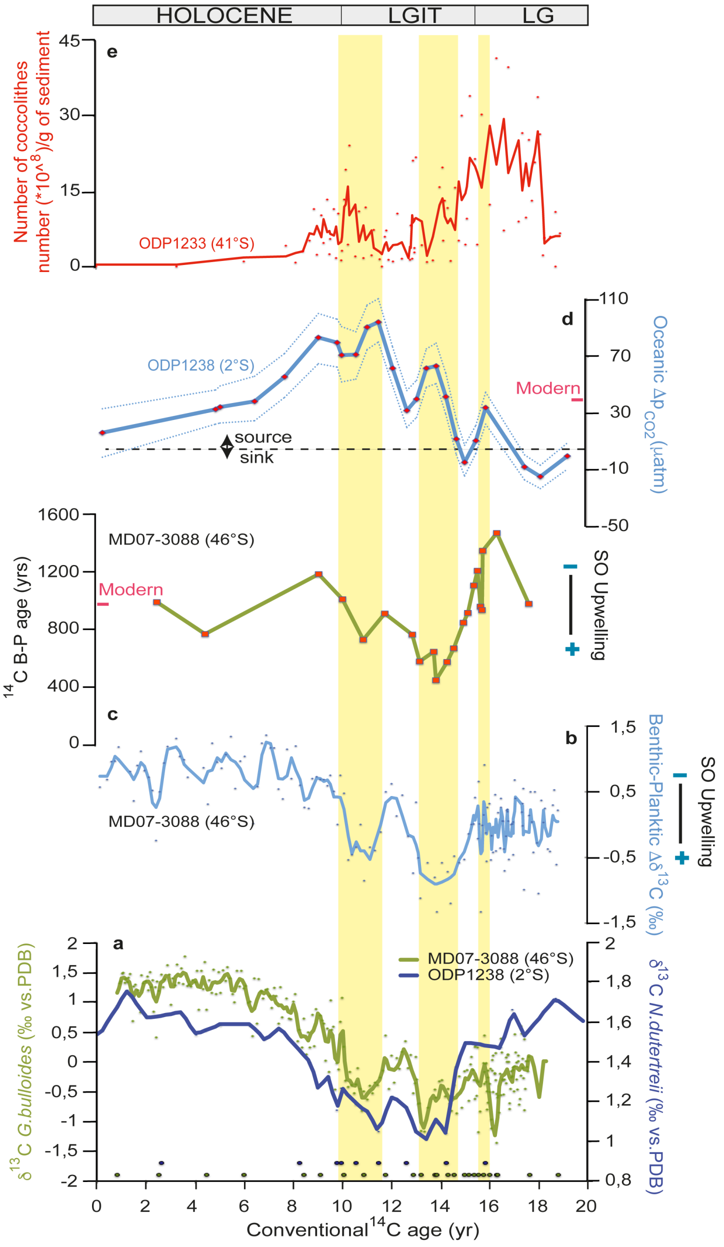

Supplementary Figure 1: Common age model for SEP cores MD07-3088 and ODP 1233 as well as EEP core ODP 1238 vs. conventional ^{14}C age. (a) Comparison between δ^{13}C *G. bulloides* (46°S) and δ^{13}C *N. dutertreii* (2°S) vs conventional ^{14}C age. Smoothed curve for core MD07-3088 using a 3 points average, circles correspond to AMS ^{14}C dating points along both cores. (**b)** Paired benthic and planktonic foraminifera $\Delta\delta^{13}$C in core MD07-3088[1]; smoothed curve using a 3 points average. (**c**) Paired benthic and planktonic foraminifera radiocarbon age difference in core MD07-3088[1]. (**d**) Oceanic ΔpCO_2 (µatm) of ODP 1238[2]. **(e)** Number of coccoliths at site ODP 1233 (/g of sediment)[3]; smoothed curve using a 3 points average.

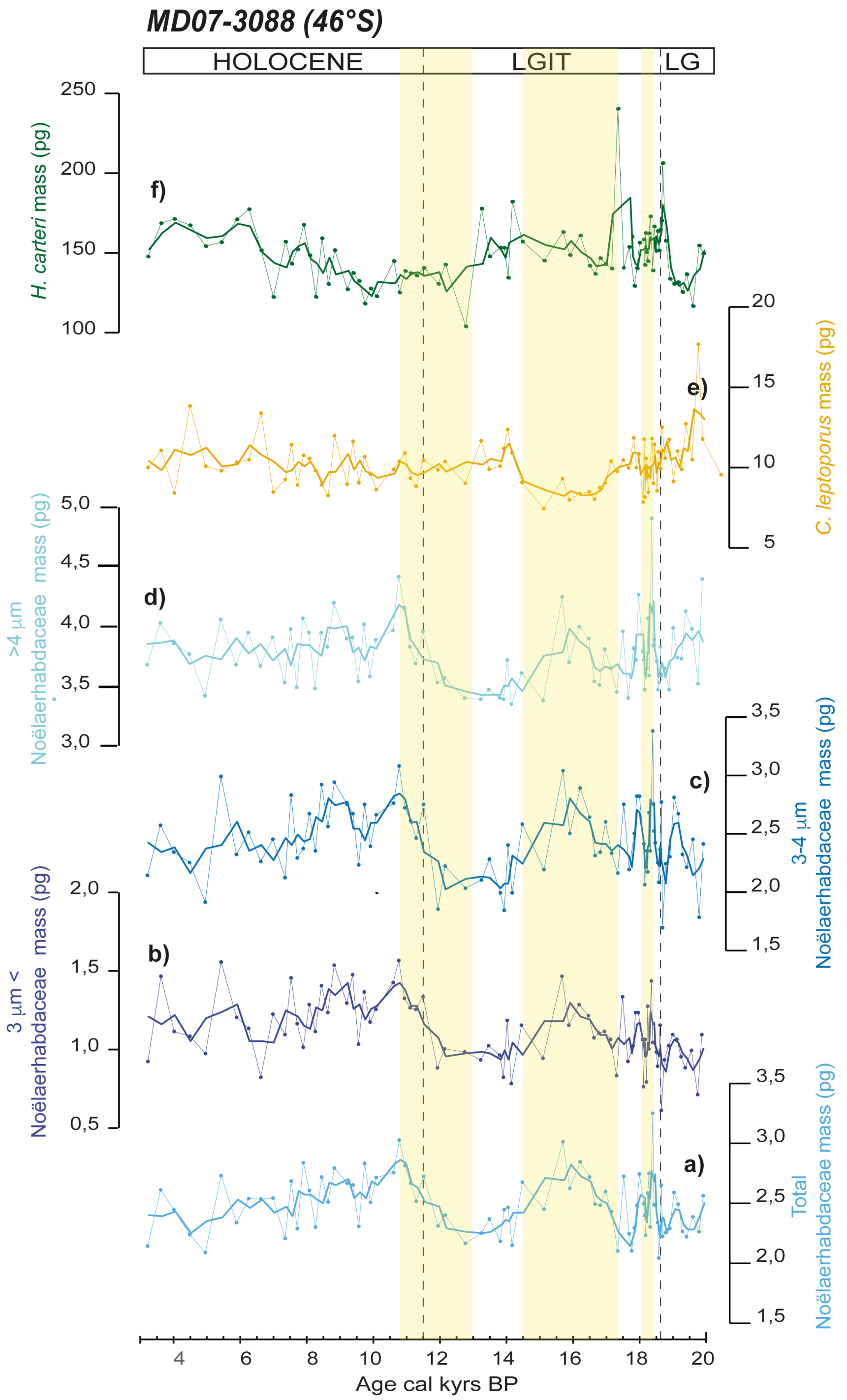

Supplementary Figure 2: **Variations of coccolith and main species/morphotype mass at site MD07-3088 over the last 3–20 ka. (a-d)** Masses of total, <3µm, 3-4 µm, and > 4 µm Noëlaerhabdaceae **(e)** Mass of *C. leptoporus*; **(f)** Mass of *H. carteri*. The error bar is of +/- 3%. Yellow shading highlight enhanced SO upwelling during the last deglaciation in phase with increasing Noëlaerhabdaceae mass regardless of the size-related morphotypes, as well as decreasing or relatively low *C. leptoporus* and *H. carteri* masses. Such patterns highlight increasing [CO_{2aq}] in surface waters at site MD07-3088 during SO upwelling.

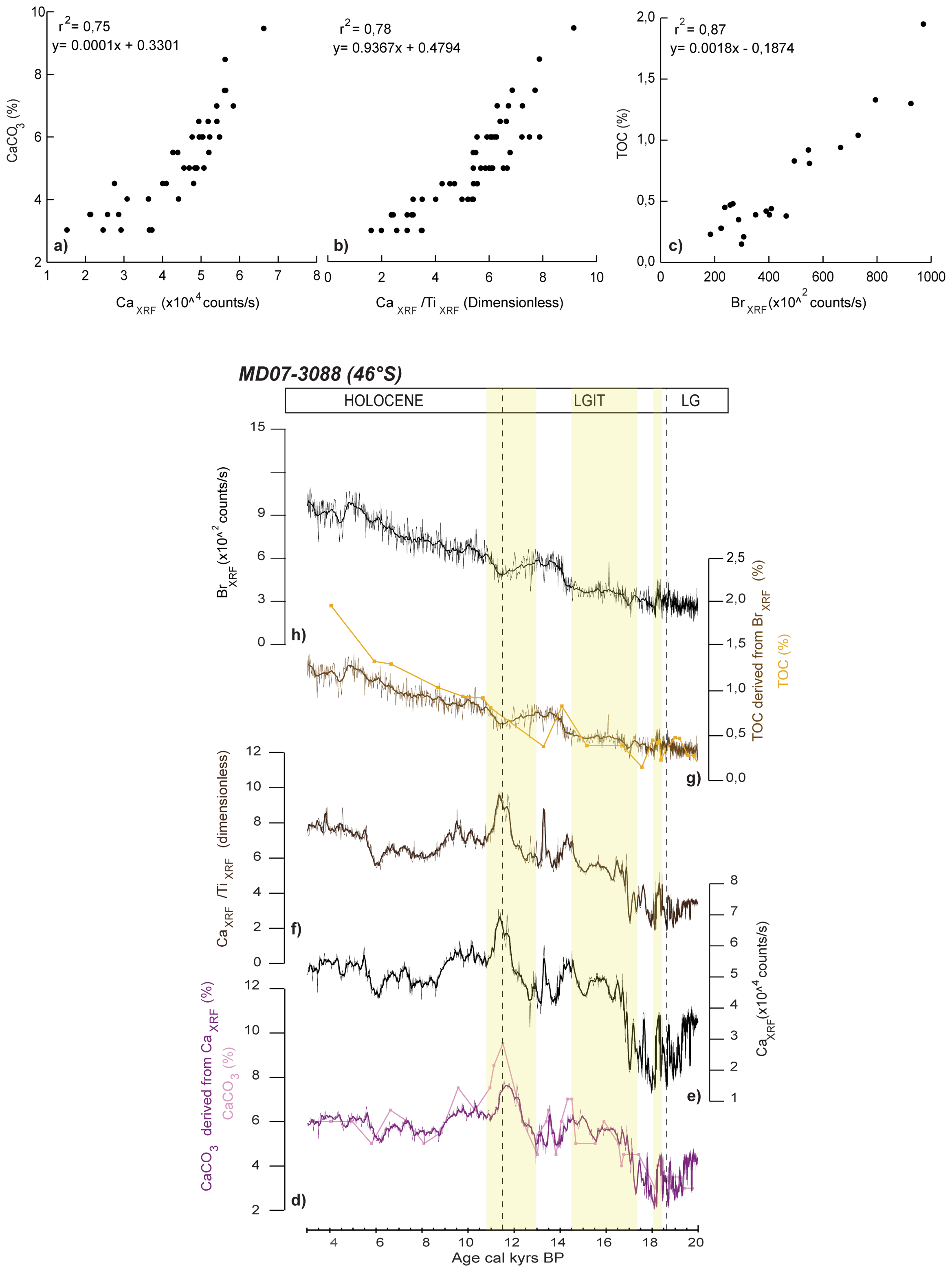

Supplementary Figure 3: Comparison of $CaCO_3$, Ca_{XRF}, and Ca_{XRF}/Ti_{XRF} records as well as TOC and Br records at site MD07-3088 over the last 3–20ka. (a-b) $CaCO_3$ is strongly correlated with Ca and Ca/Ti (that yields a geochemical record of biogenic carbonate free of

biases due to terrigenous inputs[4]). Ca may be converted into $CaCO_3$ following the linear relationship documented in **(a)** (y = 0,0001x + 0,3301), with an error bar of +/- 0.83% to highlight its short-term evolution over the last 3–20 ka. The significant correlation coefficient value between TOC (%) and Br (counts/s) **(c)** shows that Br may be related to TOC. According to the linear relationship documented in **(c)** (y = 0,0018x-0,1874), Br intensity is converted into organic carbon contents with an error bar of +/- 0.23% to determine its rapid evolution over the last 3–20 ka. Yellow shading characterizes enhanced SO upwelling during the last deglaciation, in conjunction with increased $CaCO_3$ contents, as well as increased TOC (HS1) or slowly decreasing but relatively high TOC (YD) thus, highlighting the central role of enhanced CCP in subduing the BCP during these time intervals.

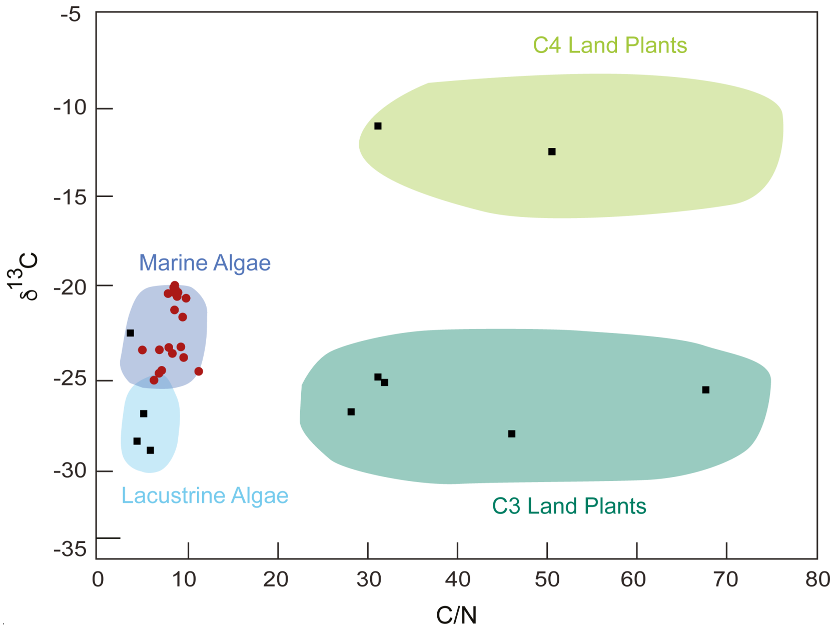

Supplementary Figure 4: Origin of the TOC. $\delta^{13}C$ and C/N values of the organic carbon (solid black circles) from the MD07-3088 core plotted against marine algae, lacustrine algae C3 and C4 land plants values[5]. Solid black squares symbolize compiled data from Ref. 5. Solid red circles represent data from this study.

Supplementary References

1. Siani, G., Michel, E. De-Pol Holz, R., DeVries T., Lamy, F., Carel, M., Isguder, G., Dewilde F., Lourantou A. Carbon isotope records reveal precise timing of enhanced Southern Ocean upwelling during the last deglaciation. Nature Communications 4, 2758. doi: 10.1038/ncomms3758 (2013).

2. Martinez –Boti, M. A., Marino, G., Foster, G. L., Ziveri, P., Henehan, M. J., Rae, J. W. B., Mortyn, P. G., Vance, D. Boron isotope evidence for oceanic carbon dioxide leakage during the last deglaciation. *Nature* **518**, 219-222 (2015).

3. Saavedra-Pellitero, M., Flores, J. A., Lamy, F., Sierro, F. J., Cortina, A. Coccolithophore estimates of paleotemperature and paleoproductivity changes in the southeast Pacific over the past ~27 kyr. *Paleoceanography*, **26**, PA1201, doi:10.1029/2009PA001824 (2011).

4. Siani, G., Colin, C., Michel, E., Carel, M., Richter, T., Kissel, C., Dewilde, F. Late Glacial to Holocene terrigenous sediment record in the Northern Patagonian margin: Paleoclimate implications. *Paleogeogr. Paleoclimatol. Paleoecol.* **297**, 26–36 (2010).

5. Meyers, P.A. Preservation of elemental and isotopic source identification of sedimentary organic matter. *Chemical Geology* **114**, 289–302 (1994).
